# Value-Added Use of Invasive Plant-Derived Fibers as PHBV Fillers for Biocomposite Development

**DOI:** 10.3390/polym13121975

**Published:** 2021-06-16

**Authors:** Xiaoying Zhao, Tolulope Lawal, Mariane M. Rodrigues, Talen Geib, Yael Vodovotz

**Affiliations:** 1 Department of Food Science and Technology, College of Food, Agricultural, and Environmental Sciences, The Ohio State University, 2015 Fyffe Road, Columbus, OH 43210, USA; zhao.1630@osu.edu; 2 Department of Materials Science and Engineering, College of Engineering, The Ohio State University, 2041 College Road, Columbus, OH 43210, USA; lawal.10@buckeyemail.osu.edu; 3 Department of Food Engineering, School of Animal Science and Food Engineering, University of Sao Paulo, 225 Duque de Caxias, Pirassununga 13635-900, SP, Brazil; mariane.mendes.rodrigues@usp.br; 4 Consultant, 361 E 20th Avenue Apt A, Columbus, OH 43201, USA; talendgeib@gmail.com

**Keywords:** polymer–matrix composites (PMCs), discontinuous reinforcement, fibers, fiber/matrix bond

## Abstract

Poly(3-hydroxybutyrate-co-3-hydroxyvalerate) (PHBV) is a promising biobased, biodegradable thermoplastic with limited industrial applications due to its brittleness and high cost. To improve these properties, lignocellulosic fibers from two invasive plants (*Phalaris arundinacea* and *Lonicera japonica*) were used as PHBV reinforcing agents. Alkali treatment of the fibers improved the PHBV–fiber interfacial bond by up to 300%. The morphological, mechanical, and thermal properties of the treated fibers were characterized, as well as their size, loading, and type, to understand their impact on performance of the biocomposites. The new biocomposites had improved thermal stability, restricted crystallization, reduced rigidity, and reduced cost compared with PHBV. Additionally, these novel biocomposites performed similarly to conventional plastics such as polypropylene, suggesting their potential as bio-alternatives for industrial applications such as semirigid packaging and lightweight auto body panels.

## 1. Introduction

Fiber-reinforced polymer composites consist of a polymer matrix and embedded fiber fillers [1,2]. Fiber inclusion serves to increase strength and stiffness and improve thermal conductivity [2]. Traditional polymer composites are reinforced with synthetic fibers [2] and have wide applications in automotive, sport, marine, aircraft, and other industries [1,2]. Plant fibers, or lignocellulosic fibers, have been attracting increased interests as “green” substitute for synthetic fibers due to their natural abundance, renewability, biodegradability, low cost, light weight, acceptable mechanical properties, and good thermal insulation properties [3,4,5,6]. Some plant fiber composites have found applications in packaging, furniture, automobile, and building industries [3]. Globally, lignocellulosic fibers are abundantly available, but they must be used in a sustainable manner [7]. Value-added use of lignocellulosic fibers from underexplored resources, such as agro-food waste and invasive plant species, can help improve the long-term sustainability of the polymer composite industry.

Invasive plant species are a long-standing problem in the United States [8]. They upset ecological balance by competing with native species for light and nutrients and adversely affecting fish and wildlife habitats [9], costing the U.S. economy $120 billion annually [10]. One of the most effective mechanical methods to control invasive plants is consecutive-cutting, which generates a significant amount of lignocellulosic biomass [11,12], globally estimated as 180 × 10^9^ tons/year, with about 7–18 × 10^9^ tons remaining potentially accessible [13]. Without suitable handling, this biomass may reproduce through vegetative propagation and further invade ecosystems [11]. Value-added use of the invasive plants for polymer composite fabrication can help manage the ecological problems and encourage harvest instead of expensive control and disposal, ensuring significant environmental and economic benefits [14].

Mechanical performance of the plant fiber composites mainly depends on (i) fiber strength and modulus’ (ii) fiber microstructural parameters such as diameter, length, and aspect ratio and fiber content and alignment in the matrix; (iii) mechanical and chemical properties of the matrix; and (iv) fiber–matrix interfacial bond, i.e., effectiveness of stress transfer across the fiber–matrix interface [15]. The strength of the interfacial bond mainly depends on fiber surface energy, chemistry, and roughness, and matrix chemistry [3]. Lignocellulosic fibers are generally hydrophilic and incompatible with hydrophobic thermoplastics [16], resulting in poor interfacial bond and difficulty in mixing [16], limiting the composite performance improvement. Optimizing fiber–matrix interfacial bond through chemical treatment of fibers is the focus of much research on plant fiber composites [3].

Alkaline treatment, i.e., mercerization, is an effective method to increase fiber–matrix bond [17]. Mercerization improves fiber surface toughness, disrupts fiber hydrogen bonding, and increases the amount of cellulose exposed on fiber surface, resulting in better fiber–matrix interfacial adhesion [17]. This treatment also removes a certain amount of hemicellulose, lignin, wax, and oils on the fiber surface; depolymerizes part of the cellulose; and exposes the short-length crystallite [18,19]. The following reaction occurs during alkali treatment [20]:

(1) $$\mathrm{Fiber}\text{-}\mathrm{OH}+\mathrm{NaOH}\;\to {\;\mathrm{Fiber}\text{-}O\text{-}\mathrm{Na}}^{+}\;{+H}_{2}O$$

Distinct alkali treatment is used for different fibers to obtain optimal fiber properties, improve fiber–matrix bond, and enhanced composite performance. In addition, plant fibers have varying affinity with polymers that differ in structural/chemical properties, resulting in differences in their reinforcement effect [15]. Additionally, fiber dimension/loading can alter reinforcement depending on the specific matrix. Therefore, development of composites with different fibers and matrices requires a full understanding of the specific fiber–matrix bond and the effect of alkali treatment on the fiber–matrix bond and composite. So far, very few studies have been reported on exploring the invasive plant fiber–plastic interfacial bond, limiting the development of invasive plant fiber composites with improved performance.

Currently, the most used polymers for plant fiber composites are petroleum-based and noncompostable, such as polystyrene and polypropylene. There is an increasing interest in replacing these polymers with biobased and/or biodegradable polymers, such as polylactic acid (PLA) and poly(3-hydroxybutyrate-co-3-hydroxyvalerate) (PHBV), to develop fully “green” composites, which are promising in applications where material recycling is difficult, biodegradability is preferred, and the product has a short lifespan [21,22,23]. Among them, PHBV biocomposites are attracting increasing interest due to their good biodegradability, nontoxicity, and acceptable mechanical properties [24].

Our group previously developed a biobased compostable composite from PHBV and invasive plant fibers (untreated) [14,25]. The composite had improved Young’s modulus and complex viscosity, but decreased tensile strength and tensile elongation at 10% fiber loading. Thus, improving the composite strength through a better fiber–matrix bond accomplished by fiber chemical treatment is a logical next step. In the present work, we studied the effect of alkali treatment on the mechanical properties of invasive plant fibers and fiber–PHBV interfacial bond. We also explored the relationship between fiber–matrix bond and composite properties and the effect of fiber type, loading, and size on composite performance. This study will inform future research on value-added use of invasive plants from different resources for green composite manufacture.

## 2. Materials and Methods

### 2.1. Invasive Plant Collection, Cleaning, and Fiber Extraction

**Figure 1:**
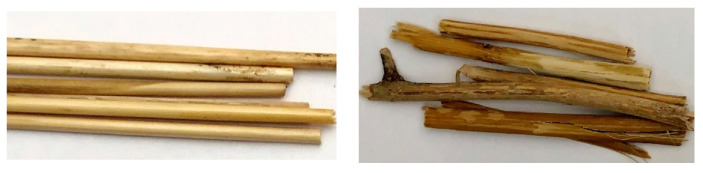
Pretreated invasive plant fibers (**left**: reed canarygrass, **right**: honeysuckle).

Two invasive plants, *Phalaris arundinacea*, which is referred as reed canarygrass (RC) in this paper, and *Lonicera japonica*, which is referred to as honeysuckle (HS) in this paper, were collected from Olentangy River Wetland, Columbus, Ohio, USA. The leaves were removed, and unskinned stems were cleaned with water and dried to constant weight (Figure 1). Fiber bundles with a diameter of 100–500 μm and length of 2–8 cm were extracted using a blade. PHBV with approximate 2 mol% hydroxyvalerate (HV) content and a molecular weight (Mw) of around 240 kDa [26] was purchased from Tianan Biological Material Co. (Ningbo, China). 

### 2.2. Alkali Treatment of the Fibers

The fibers were soaked in NaOH solutions (concentrations of 2, 6, 10 wt.%) for a specific period (1, 3, 5 h) at room temperature. The treated fibers were washed to remove residual NaOH and dried at 80 °C to constant weight. The optimal NaOH treatment was determined by evaluating fiber tensile strength, tensile elongation, and fiber–PHBV interfacial bond.

### 2.3. Morphological Characterization of the Fibers

The fibers were coated with a 10 nm layer of gold using a Cressington 108 sputter coater (Watford, UK) and visualized using a Quanta 200 (FEI Inc., Hillsboro, OR, USA) SEM.

### 2.4. Mechanical Characterization of the Fibers

Tensile properties were tested according to ASTM C1557-14 using an Instron 5542 (Instron Corp., Norwood, MA, USA). The testing rate for RC and HS fibers were 5 and 8 mm/min, respectively, to provide a test time to specimen fracture within 30 s. At least 15 specimens were tested for each sample.

### 2.5. Measurement of the Fiber–Matrix Interfacial Bond

For sample preparation, fiber was embedded in PHBV with a 0.5–3 mm embedding length [27,28] by inserting the fiber into a pre-melted PHBV pellet followed by being cooled at room temperature. An Instron 5542 was used to measure the maximum force required to pull the fiber out from PHBV at 5 mm/min. At least 15 specimens for each sample were tested. The interfacial shear strength (IFSS), i.e., fiber–matrix interfacial bond IFSS, was calculated as below [29]:

(2) $$\;\tau =\frac{F_{\max}}{d_{f}\;\cdot \;\pi \;\cdot l_{\mathrm{ef}}\;}=\frac{F_{\max}}{P_{f}\;\cdot {\;l}_{\mathrm{ef}}}$$

τ: IFSS between the fiber and PHBV, F_max_: maximum force required to pull the fiber out from PHBV, d_f_: fiber dimeter, P_f_: fiber perimeter, calculated using a Stream image analysis software v.2.4.2 from the fiber cross sectional area measured by a stereomicroscope (Olympus, Waltham, MA, USA), l_ef_: fiber embedding length.

### 2.6. Fabrication of PHBV/Invasive Plant Biocomposite Through Extrusion

**Figure 2:**
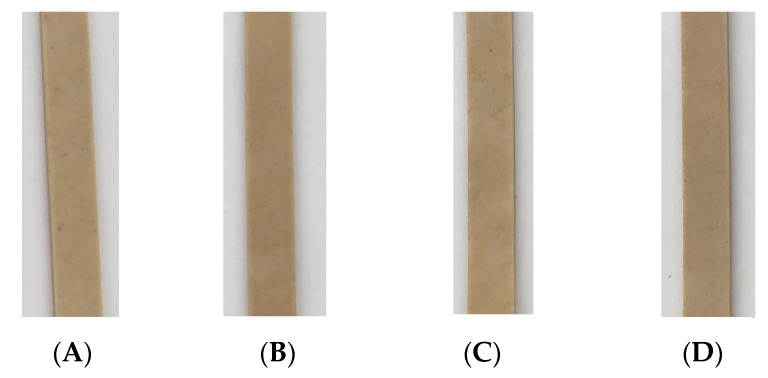
Representative PHBV/invasive plant fiber composite samples: (**A**) PHBV-R-IV-10%, (**B**) PHBV-R-IV-10%-control, (**C**) PHBV-H-IV-10%, (**D**) PHBV-H-IV-10%-control.

Untreated invasive plants were ground using a Thomas Wiley-4 mill (Thomas Scientific, Swedesboro, NJ, USA) and sieved to obtain particles with four sizes (Table 1). Fiber particles after optimal NaOH treatment were compounded with PHBV using a twin-screw extruder (Leistritz, Somerville, NJ, USA) at a reverse temperature of 185–145 °C and a rate of 30 rpm (Table 1) to obtain PHBV/invasive plant fiber biocomposites (Figure 2, Table 2). The To study the effect of the fiber loading on the composite performance, honeysuckle composites (fiber particle size 297–425 μm) with 5–20 % *v*/*v* fiber loading were prepared (Table 2).

### 2.7. Thermogravimetric Analysis (TGA) of the Fibers and Composites

Samples were heated from room temperature to 600 °C at 20 °C/min using a TGA 550 (TA Instruments, New Castle, DE, USA). Onset (T_o_) and peak (T_p_) degradation temperatures were obtained from the TGA and DTG (derivative thermogravimetry) thermograms using a TRIOS software (v4.1.1.33073).

### 2.8. Thermal Analysis of the Fibers and Composites Using Differential Scanning Calorimetry (DSC)

Samples were heated from room temperature to 185 °C at 10 °C/min, held for 2 min, cooled to −85 °C at 10 °C/min, held for 4 min, and heated to 200 °C at 10 °C/min using a DSC2500 (TA Instruments, New Castle, DE, USA). Glass transition temperature (T_g_), melting temperature (T_m_), enthalpy (ΔH_m_), and degree of crystallinity (X_c_) were determined from the second heating scans. X_c_ was obtained by dividing the ΔH_m_ by the enthalpy of a theoretically 100% crystalline PHBV (146 J/g) [24].

### 2.9. Mechanical Characterization of the Composites

Tensile testing was conducted according to ASTM D 638-08 using an Instron 5542, with a 25.4 mm grip distance at 5 mm/min, using dumbbell-shaped specimens (Type V) prepared from compression molding. At least five specimens were tested for each sample.

### 2.10. Statistical Analysis

Statistical analysis was performed using JMP 10.0 (Marlow, Buckinghamshire, UK). Significant difference (*p* < 0.05) was determined using ANOVA and Tukey HSD.

## 3. Results and Discussion

### 3.1. Alkali Treatment Improved Fiber Surface Roughness and Fiber Bundle Separation

**Figure 3:**
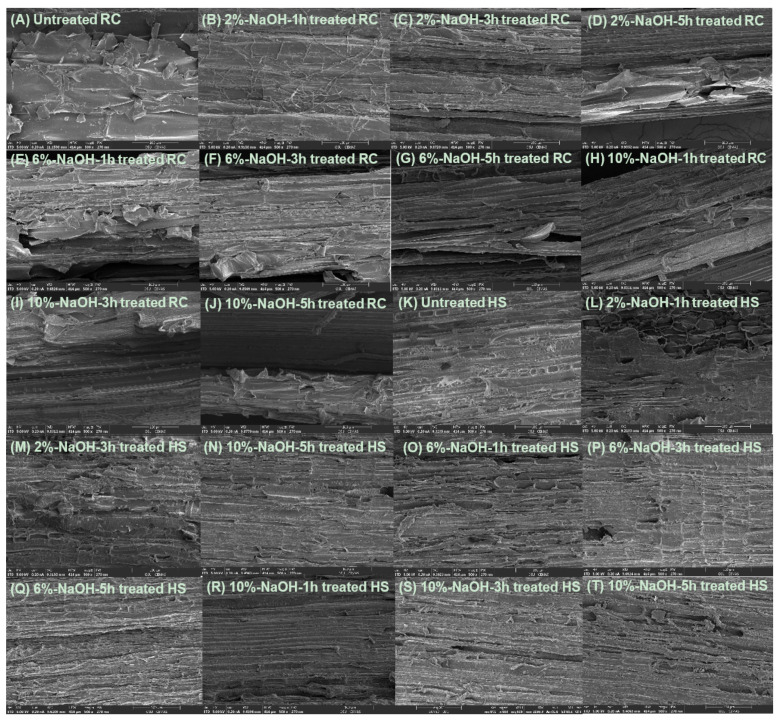
SEM images of untreated and NaOH-treated RC and HS fibers. The scale of the SEM images is 100 µm.

Untreated RC fibers (Figure 3A) were covered with parenchymatous pith, a soft and spongy tissue in the stems of vascular plants composed of parenchyma cells which mainly consist of cellulose, hemicellulose, and lignin [30]. Cellulose from pith has lower degree of polymerization and lower strength than that from fiber bundles. Alkali treatment removed the pith and revealed fiber bundles with aggregated monofilaments (Figure 3B–J), which are bound together by hemicellulose/lignin in the interfibrillar region [31]. The fiber surface cleanness, roughness, and bundle separation increased with increasing NaOH concentration and treatment time (Figure 3B–J). The fiber composition change caused by the alkali treatment was further substantiated by the TGA analysis (Section 3.2). Similarly, HS fibers after alkali treatment had a cleaner and rougher surface with more obvious bundle separation (Figure 3K–T). The surface improving trend was also depending on NaOH concentrations and treatment time. The removal of the pith, termed de-pithing, and part of the hemicellulose/lignin can help improve fiber mechanical performance [30] (Section 3.3) and fiber–matrix interlocking [31] (Section 3.4).

### 3.2. Alkali Treatment Removed Hemicellulose and Lignin in the Fiber

**Figure 4:**
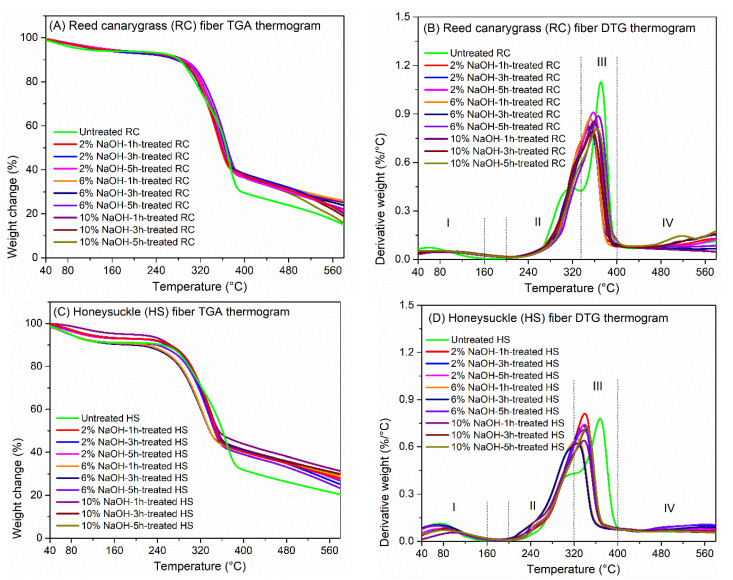
(**A**) Thermogravimetric analysis (TGA) thermograms of the untreated and alkali-treated reed canarygrass fibers. (**B**) Derivative thermogravimetric (DTG) thermograms of the untreated and alkali-treated reed canarygrass fibers. (**C**) TGA thermograms of the untreated and alkali-treated honeysuckle fibers. (**D**) DTG thermograms of the untreated and alkali-treated honeysuckle fibers.

The thermal degradation of the untreated RC and HS fibers both occurred in four stages (Figure 4A–D). For the untreated RC fiber (Figure 4A,B), the first stage (I) of weight loss (6.0%) occurred from room temperature to 160 °C, with a T_p_ of 60 °C mainly due to moisture vaporization [32]. Between 150 and 200 °C, the RC fiber presented thermal stability (weight loss < 1%), suggesting that 200 °C is the thermally stable and maximum processing temperature of RC fibers. The second stage (II) of weight loss (24.5%) of the untreated RC fiber occurred from 200 to 335 °C, with a T_p_ of 319 °C, and was likely due to hemicellulose thermal depolymerization and glycosidic link breakdown in cellulose [33]. The third stage (III) of weight loss (39.8%) of the untreated RC fiber occurred from 335 to 400 °C, with a T_p_ of 372 °C, and was attributed to the degradation of the cellulose and a small part of the lignin. This peak cellulose-loss degradation temperature is higher than that of other natural fibers such as bamboo (321 °C), Prosopis juliflora (331.1 °C), hemp (308.2 °C), kenaf (309.2 °C), and jute fibers (298.2 °C) [34]. The degradation of the cellulosic components takes place mostly in the amorphous regions (hemicellulose) [35] between 200–400 °C and causes reactions such as dehydration, decarboxylation, and decarbonylation and breaking of C-H, C-O, and C-C bonds [36]. The fourth thermal degradation stage (IV) of the untreated RC fiber above 400 °C could be mainly due to lignin degradation since it is the most difficult component to decompose in plant fiber due to its complex composition of aromatic rings with various branches [37]. Lignin usually starts to degrade at around 200–350 °C at a very low weight loss rate and may reach up to 600–700 °C or even higher [38].

Similarly, the untreated HS fibers had four thermal degradation stages (Figure 4C,D), i.e., stage I from room temperature to 160 °C (weight loss 5.4%, T_p_ = 55 °C), stage II from 200 to 320 °C (weight loss 24.3%, T_p_ = 312 °C), stage III from 320 to 400 °C (weight loss 25.3%, T_p_ = 363 °C), and stage IV above 400 °C, with a thermally stable range of 160–200 °C (weight loss 0.7%). The untreated HS fiber had similar a weight loss and T_p_ as the untreated RC fiber in both stage I and II, suggesting the two fibers contain similar concentrations of moisture and hemicellulose. Meanwhile, HS fiber lost less weight in stage III and more weight in stage IV, indicating it had less cellulose and more lignin than RC fiber. These results are consistent with previous findings, where RC fiber was composed of ~28% cellulose, ~22% hemicellulose, and 14% lignin, while HS fiber was composed of more lignin (~25%) and similar content (~25%) of hemicellulose [14,39]. The difference in the compositions of the two fibers results in their different mechanical performance, reactions to alkali treatment, and interactions with PHBV matrix, which will be discussed later.

Both the alkali-treated RC and HS fibers (Figure 4B,D) had a much weaker hemicellulose-loss peak (T_p_ in stage II) compared with the untreated RC and HS fibers, indicating the removal of part of the hemicellulose upon NaOH treatment. The hemicellulose removal efficiency increased with increasing NaOH concentration but did not depend on treatment time. Additionally, the cellulose weight-loss peak (T_p_ in stage III) shifted toward lower temperature by 10–20 °C upon NaOH treatment, suggesting the removal of part of the lignin upon NaOH treatment, since some lignin degraded at higher temperature than cellulose. The removal of the amorphous hemicellulose and lignin could result in increased fiber crystallinity. This was supported by the fact that the alkali-treated fibers had less moisture loss in stage I than the untreated fibers, as moisture was strongly held within the tightly packed (highly crystalline) structure of the fibers [35]. The improved fiber crystallinity could also facilitate improved mechanical performance [40] (Section 3.3). Finally, NaOH treatment did not affect the thermally stable range of the two fibers, indicating the treated fibers can be used in composite manufacturing with a maximum temperature of 200 °C.

To summarize, both the untreated RC and HS fibers had four thermal degradation stages, caused mainly by moisture evaporation, hemicellulose degradation, degradation of cellulose and part of the lignin, and lignin degradation, respectively. Alkali treatment removed part of the hemicellulose and lignin, thus improving the mechanical performance of the fibers and fiber–PHBV interfacial bond (Section 3.3 and Section 3.4).

### 3.3. Alkali Treatment Improved Fiber Tensile Strength

**Figure 5:**
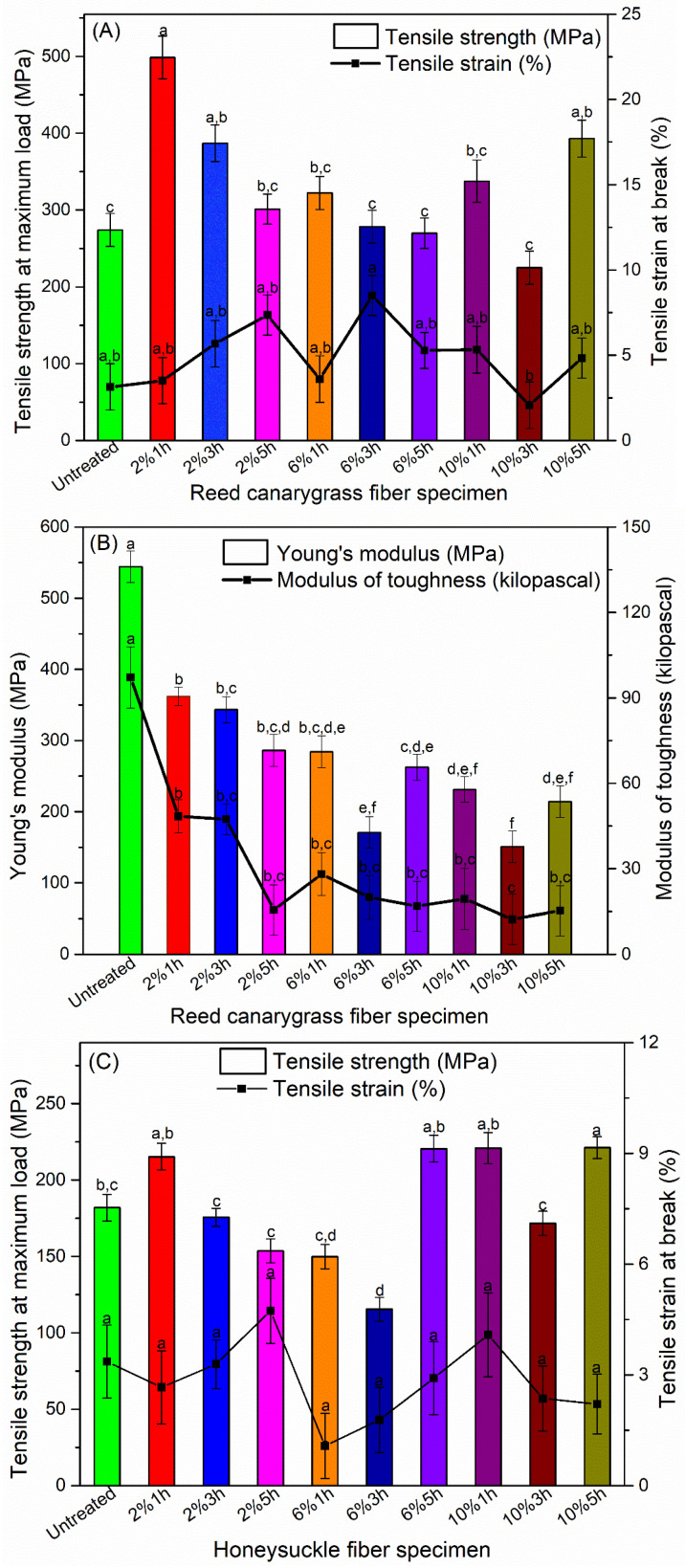

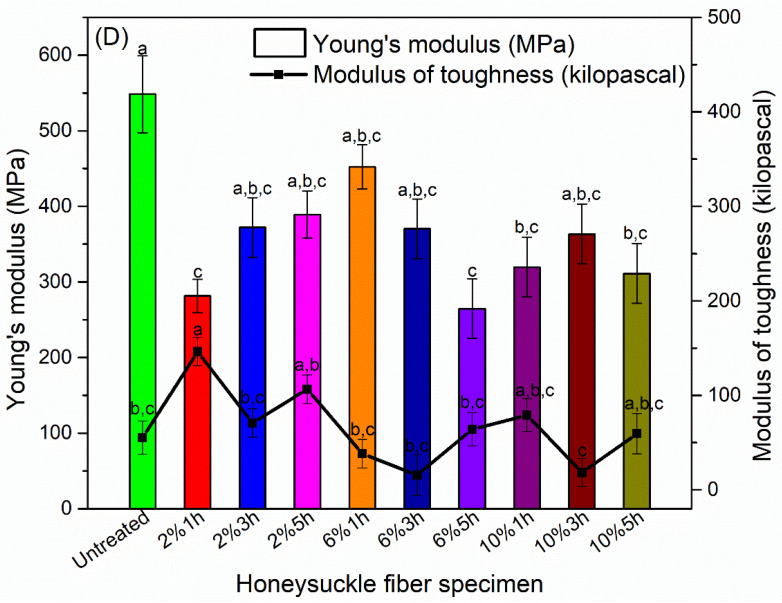
(**A**) Tensile strength and tensile strain of untreated and alkali-treated RC fibers. (**B**) Tensile modulus and modulus of toughness of untreated and alkali-treated RC fibers. (**C**) Tensile strength and tensile strain of untreated and alkali-treated HS fibers. (**D**) Tensile modulus and modulus of toughness of untreated and alkali-treated HS fibers. Labels of the x axis represents treatment conditions, i.e., A % Bh represents fiber specimen was soaked in A wt.% NaOH aqueous solution for B hour. Means showing different letters are significantly different at α = 0.05. The modulus of toughness was calculated as the area under the stress–strain curve up to the fracture point.

NaOH treatment (2% 1 h, 2% 3 h, 10% 5 h) improved RC fiber tensile strength from 274 to 387–499 MPa but did not significantly affect its tensile strain (Figure 5A). This is in consistent with previous research on alkali-treated plant fibers [41,42,43,44,45]. The improvement in the tensile strength is probably due to the alkali treatment removing the amorphous hemicellulose/lignin in the interfibrillar region, which acts as the supportive matrix for cellulose microfibrils and is more sensitive to alkali than cellulose [41,42,46,47], as observed in the SEM and TGA results. Their removal released the internal strain of the fibrils, caused the formation of new hydrogen bonds between cellulose chains, and facilitated fibril rearrangement along the direction of tensile force [48], resulting in increased fiber packing density and reduced void content in the fibers [41,42]. Therefore, when stretched, the rearranged fibrils developed higher stress due to their better load sharing [48]. The maximum increase in the tensile strength of the RC fiber was obtained by the 2%-1h-NaOH treatment. Higher NaOH concentration or longer treatment time did not further improve the tensile strength, probably due to cellulose depolymerization and fiber delignification [49,50].

RC fiber modulus (Figure 5B) decreased up on alkali treatment and is similar to that of the NaOH-treated oil palm empty fruit bunch fiber (~265 MPa) [51]. The decreased modulus was probably due to the decrease in the fiber stiffness caused by the degradation of the primary cell wall and removal of the surface impurities such as wax and gum [52]. Similar observations were reported in previous research on curaua, prosopis juliflora, and century fibers after alkali treatment [41,52]. NaOH treatment also reduced the toughness of the RC fiber, likely due to the removal of the amorphous hemicellulose, which reduced the capacity of the fibers for absorbing energy when stretched [52].

The tensile strength of the HS fibers increased from 182 to 221 MPa upon a 10%-5h-NaOH treatment, while the tensile strain had no significant change after alkali treatment (Figure 5C). Like RC fibers, the improved tensile strength of the NaOH-treated HS fibers was partially due to the increased cellulose crystallinity, which was caused by the removal of the amorphous hemicellulose and part of the lignin. However, HS fibers required much higher NaOH concentration (10%) and longer treatment time (5 h) than RC fibers for improved tensile strength. This is probably because, as a wood fiber, HS fiber has much more lignin (~25%) than RC fiber (~14%) [14,39]. Lignin is less sensitive to alkali than hemicellulose and therefore requires higher NaOH concentration and longer soak time to be removed from the fiber matrix [41,42,46,47]. The removal of the lignin and hemicellulose in the wood fiber decreases the distance between the microfibrils and causes microfibril aggregation in the fiber, which results in increased cellulose crystallinity and crystallite size [53], and these factors contributes to improved fiber strength [53]. According to previous research, high NaOH concentration (>10%) or longer treatment time (>6 h) caused partial dissolution of cellulose crystalline region and resulted in weakened mechanical properties [53,54].

The tensile modulus of HS fibers reduced after alkali treatment (Figure 5D), indicating lower fiber rigidity. It has been reported that alkali treatment caused contraction of cellulose microfibrils due to an entropy increase in the less ordered regions along the microfibril direction and the transformation of the crystalline structure [55]. These factors, together with the cell wall dissolution caused by the alkali treatment, contributed to the decreased fiber modulus. Unlike RC fiber, HS fiber had improved toughness after alkali treatment (2% 1 h), which may be due to a mild alkali treatment removing partial lignin and hemicellulose and improving the molecular mobility of the cellulose chains but not significantly increasing the cellulose crystallinity. As a result, the ability of the fiber to absorb energy under stretching, i.e., modulus of toughness, was improved [53,54].

To summarize, NaOH treatment (2% 1 h, 2% 3 h, and 10% 5 h) improved RC fiber tensile strength, decreased fiber modulus and toughness, and did not significantly affect the tensile strain. For HS fibers, a 10%-5h-NaOH treatment improved the tensile strength, and a 2%-1h-NaOH treatment improved fiber toughness, while all the NaOH treatment decreased fiber modulus and did not significantly affect the tensile strain. Fibers with improved strength and reduced modulus are preferred to be used as reinforcing fillers for brittle biobased plastics such as PHBV, which has a much lower tensile strength (35–40 MPa) [24] and a much higher tensile modulus (2000–3000 MPa) [56].

### 3.4. Alkali Treatment Improved Fiber–PHBV Interfacial Bond

**Figure 6:**
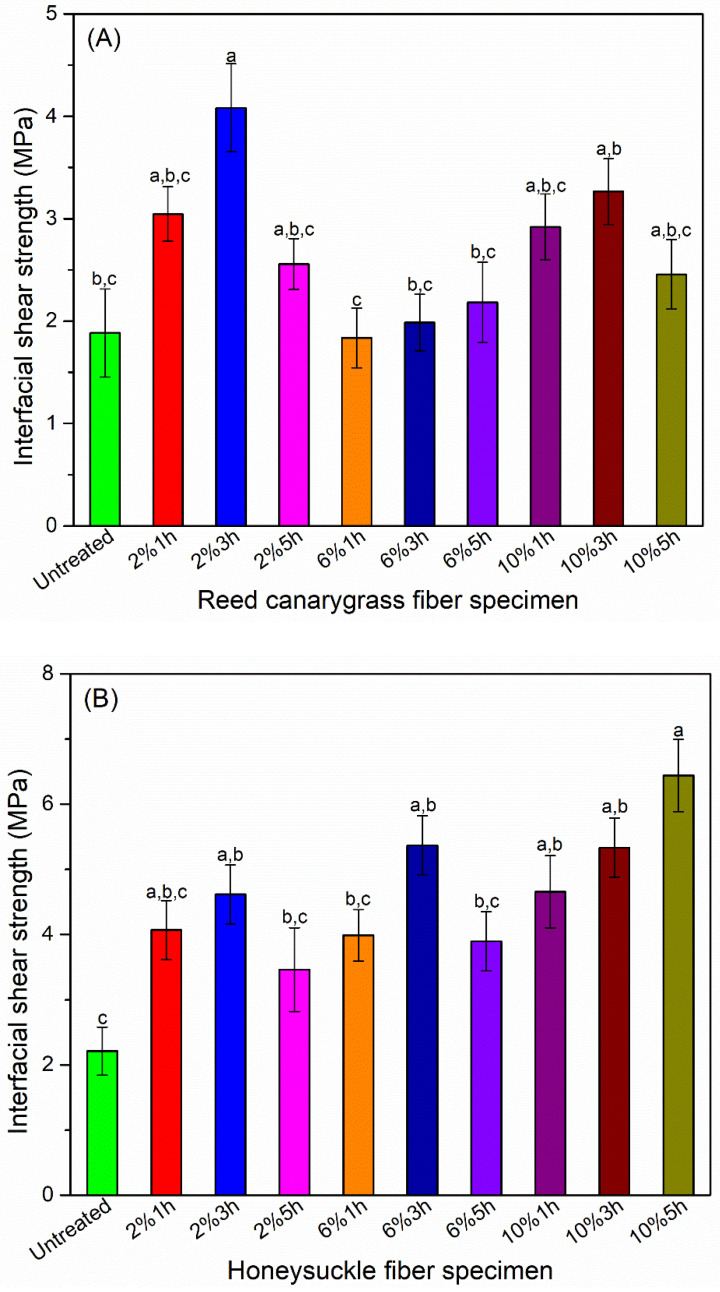
Effect of NaOH treatment on the interfacial shear strength of PHBV/fiber system: (**A**) Interfacial shear strength (IFSS), which represents the interfacial bonding between the RC fiber and PHBV matrix. (**B**) Interfacial shear strength between HS fiber and PHBV matrix. Labels of the x axis represents treatment conditions, i.e., A% Bh represents fiber specimen was soaked in A wt.% NaOH aqueous solution for B hour. Means showing different letters are significantly different at α = 0.05.

The interfacial shear strength (IFSS) of untreated RC fiber and PHBV was low (1.9 ± 0.4 MPa, Figure 6A), due to the incompatibility between the hydrophilic fiber and the hydrophobic PHBV and the existence of the impurities on the fiber surface. A 2%-3h-NaOH treatment improved the fiber–matrix interfacial bond to 3.3 ± 0.3 MPa. This value is consistent with that of the previously reported alkali-treated natural fiber-polyester [57], e.g., the IFSS of oil palm, coir, and sunhemp fibers in a polyester resin was 0.37–1.39, 0.32–1.48, and 4.34 MPa, respectively [57]. Alkali treatment improves fiber–matrix bond mainly in two ways: (i) By removing the impurities, hemicellulose, and lignin, alkali treatment reduces fiber diameter, increases the aspect ratio and the surface area of the fibers available for bonding with the matrix [58] and increases fiber surface roughness, leading to a better mechanical interlocking between the fiber and the matrix [59,60]. (ii) Alkali treatment increases the amount of cellulose exposed on the fiber surface and therefore increases the number of possible reaction sites such as hydroxyl groups on the fiber surface and results in better fiber wetting [59,60]. High NaOH concentrations (6–10%) had negligible improvements in the RC fiber–PHBV bond, probably due to fiber fibrillation, which caused poor adhesion with PHBV [61].

Different from the RC fiber, HS fiber required higher NaOH concentration (10%) and longer treatment time (5 h, Figure 6B) to improve its interfacial bond with PHBV. As discussed previously, this is likely due to HS fiber having higher amounts of lignin, which required harsher alkali treatment to roughen the fiber surface and increase the amount of the exposed cellulose. The improved fiber–matrix bond can contribute to thermal and mechanical performance of the composites, which will be discussed later.

To summarize, the optimal alkali treatment for fiber–matrix bond improvement for RC and HS fibers is 2%-3h- and 10%-5h-NaOH treatment, respectively. These treatments improved fiber tensile strength without significantly affecting the tensile strain. Fibers treated under these conditions were used for PHBV/fiber composite fabrication.

### 3.5. Fiber Addition Improved PHBV Thermal Stability

**Figure 7:**
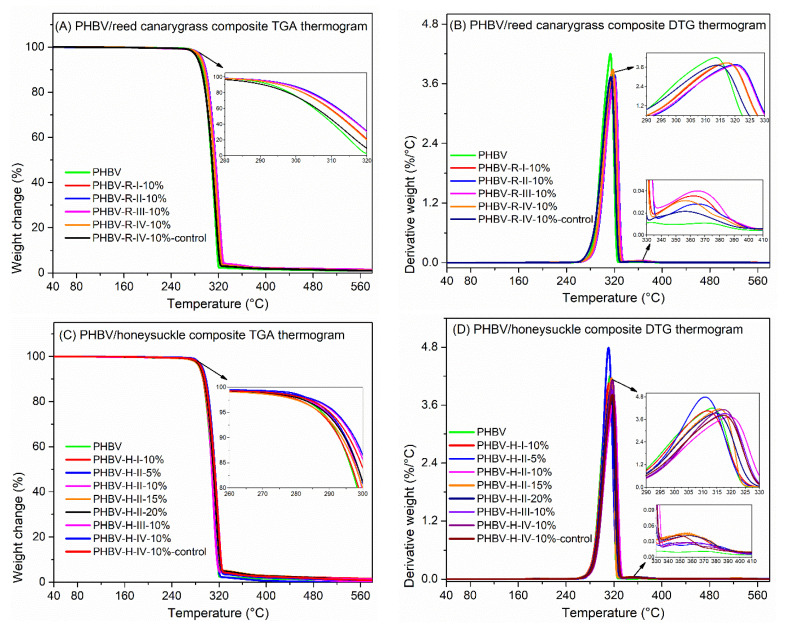
(**A**) Thermogravimetric analysis (TGA) thermograms of PHBV and its composites with RC fibers. (**B**) Derivative thermogravimetric (DTG) thermograms of PHBV and its composites with RC fibers. (**C**) TGA thermograms of PHBV and its composites with HS fibers. (**D**) DTG thermograms of PHBV and its composites with HS fibers.

The composites degraded in two stages (Figure 7A–D), reflecting the different thermal stability of PHBV and fibers. The first thermal degradation (290–330 °C) was associated with PHBV degradation, while the second stage (330–410 °C) was related to fiber degradation. Compared with PHBV, which has a T_o_ of 290.7 °C and a T_p_ of 313.2 °C, PHBV composites with alkali-treated RC fibers had a 2–5 °C increase in T_o_ and a 3–6 °C increase in T_p_, suggesting their increased thermal stability. The thermal stability increased slightly with decreasing fiber size and peaked at the sample PHBV-R-III-10%. PHBV composites with untreated RC fibers (PHBV-R-IV-10%-control) had a slightly lower T_o_ and similar T_p_ compared with PHBV, suggesting that alkali treatment improved composite thermal stability.

PHBV/HS composites with a 10% *v*/*v* fiber loading (except PHBV-H-I-10%) had a 4–5 °C increase in T_o_ and a 3–5 °C increase in T_p_ compared with PHBV (Figure 7C,D). The thermal enhancing ability of the fibers did not depend on the fiber size but decreased with increasing fiber loading, as addition of 15–20 % *v*/*v* fiber (PHBV-H-II-15% and PHBV-H-II-20%) had negligible effect on PHBV degradation. Different from the reed canarygrass fibers, alkali-treatment of the honeysuckle fibers did not significantly improve composite thermal stability.

PHBV/RC composites had a slightly higher char yield (0.8–1.4%) than PHBV (0.7%). The char yield of the composites with alkali-treated fibers did not depend on the alkali treatment conditions and were slightly higher than those with untreated fibers. PHBV/HS composites with treated and untreated fibers had a char yield of 26–29% and 13.5%, respectively, with the char yield increasing with increasing NaOH concentration.

To summarize, fiber addition improved PHBV thermal stability via increasing its T_o_ and T_p_ by 2–5 °C and 3–6 °C, respectively. RC and HS fibers had similar thermal enhancing ability but different reactions to the alkali treatment: alkali treatment improved the thermal enhancing ability of the RC fibers but had no significant effect on the HS fibers. The PHBV/HS composites had significantly increased char yield than PHBV and PHBV/RC composites, promising for flame retardant applications, as increasing char formation can limit combustible gas formation and decrease the thermal conductivity of the burning materials [62].

### 3.6. Fiber Addition Restricted PHBV Crystallization

**Figure 8:**
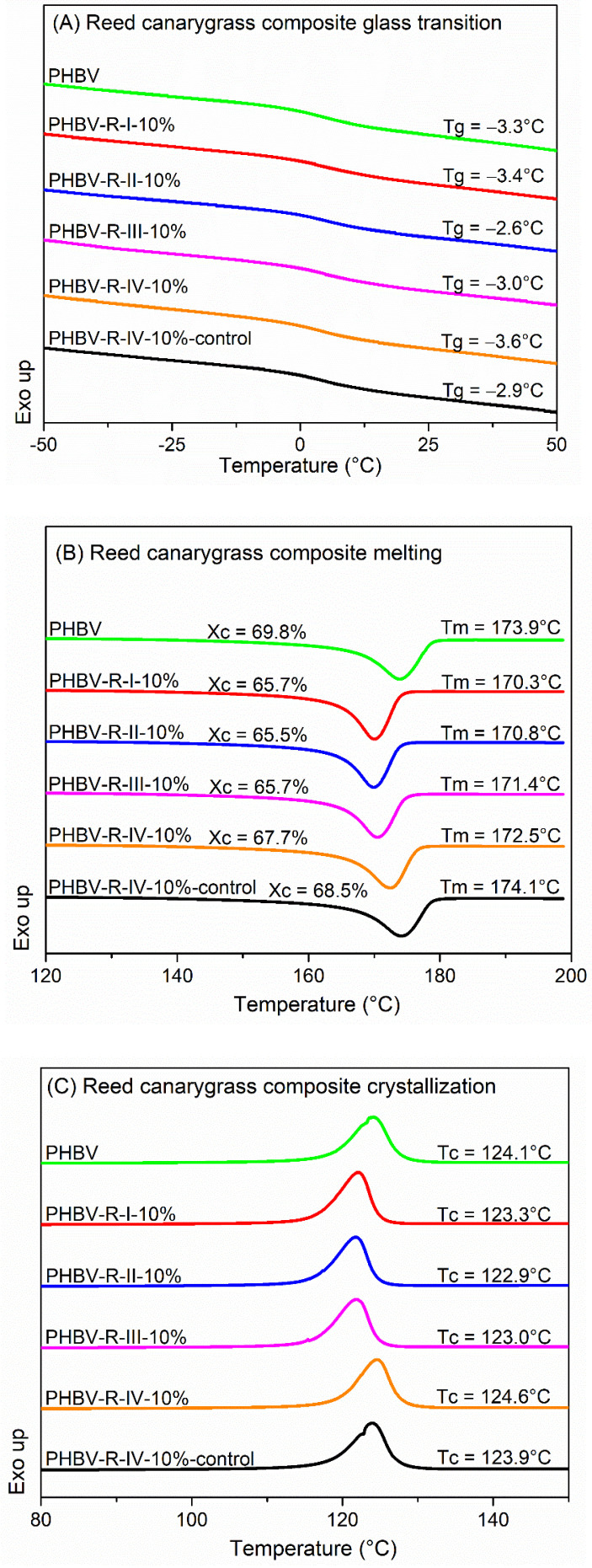

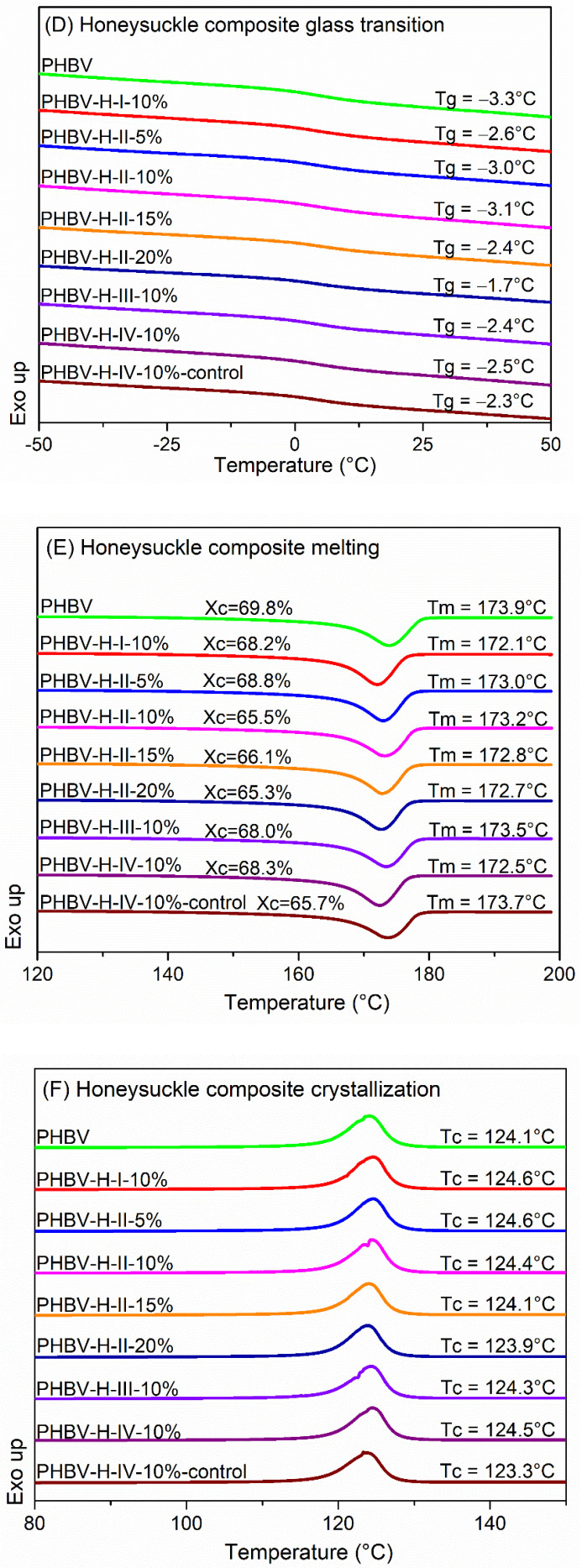
(**A**) Glass transitions of PHBV and its composites with RC fibers. (**B**) Melting transitions of PHBV and its composites with RC fibers. (**C**) Crystallizations of PHBV and its composites with RC fibers. (**D**) Glass transitions of PHBV and its composites with HS fibers. (**E**) Melting transitions of PHBV and its composites with HS fibers. (**F**) Crystallizations of PHBV and its composites with HS fibers. T_g_: glass transition temperature, T_m_: melting temperature, T_c_: crystallization temperature, X_c_: degree of crystallinity.

PHBV and its composites had a glass transition at around 5 °C (Figure 8A,D), a single melting peak at 170–175 °C (Figure 8B,E), a single nonisothermal crystallization peak at 123–125 °C (Figure 8C,F), and a degree of crystallinity ranging from 65 to 70% (Figure 8B,E). Addition of the two invasive plant fibers both increased PHBV glass transition temperature (by up to 1.6 °C), decreased the melting temperature (by up to 3.6 °C), and decreased the crystallinity (by up to 6%), indicating that PHBV crystallization ability in the composites was confined. Similar effects of other plant fibers, such as bamboo fiber, pineapple leaf fiber, and kenaf fiber, on PHBV crystallization have been reported [63,64,65,66]. The addition of the fibers restricted the mobility of the PHBV molecular chains; restrained the chain diffusion to the surface of the nuclei [65,66]; and disordered the growth of the crystals, especially at the fiber–matrix interface, causing the discontinuous introduction in the crystal structure of the matrix [63] and the formation of imperfect (e.g., thinner) crystals, resulting in decreased melting temperature and degree of crystallinity [67]. This is also reflected in the slightly decreased nonisothermal crystallization temperatures of the composites (Figure 8C,F), which suggests that the presence of the fibers delayed the crystallization process [56], i.e., fiber addition made it more difficult for the PHBV to crystallize, and as a result, the composites needed more potential energy to crystallize during the cooling scan, i.e., crystallizing at lower temperature [68].

Interestingly, the addition of the two fibers affected PHBV thermal properties differently: RC fibers with larger particle size had a more prominent effect on PHBV crystallization restriction, while HS fibers with particle size of 297–425 μm (PHBV-H-II-10%) had the most significant effect of restricting PHBV crystallization. Alkali treatment enhanced the crystallization-restricting ability of the HS fibers while having no significant effect on the RC fiber. These differences are likely caused by their different interaction with the PHBV matrix, i.e., the alkali-treated HS fibers had a stronger interfacial bond with PHBV than the alkali-treated RC fibers, as discussed in Section 3.4. Lastly, the effect of the fiber addition on restraining PHBV crystallization became more significant with higher fiber loading, as observed in composite samples PHBV-H-II-5%, -10%, -15%, and -20%.

The decreased crystallinity can help improve the processability and low-temperature flexibility of the PHBV/fiber composites but may reduce their strength and modulus (see Section 3.7). Nonetheless, the studied composites remained highly crystalline and exhibited high tensile strength comparable to some of the commercial plastic materials such as PP (22–34 MPa) and high-density polyethylene (HDPE 14.5–38 MPa) [68,69].

### 3.7. Fiber Addition Decreased PHBV Rigidity without Affecting Its Tensile Strength

**Figure 9:**
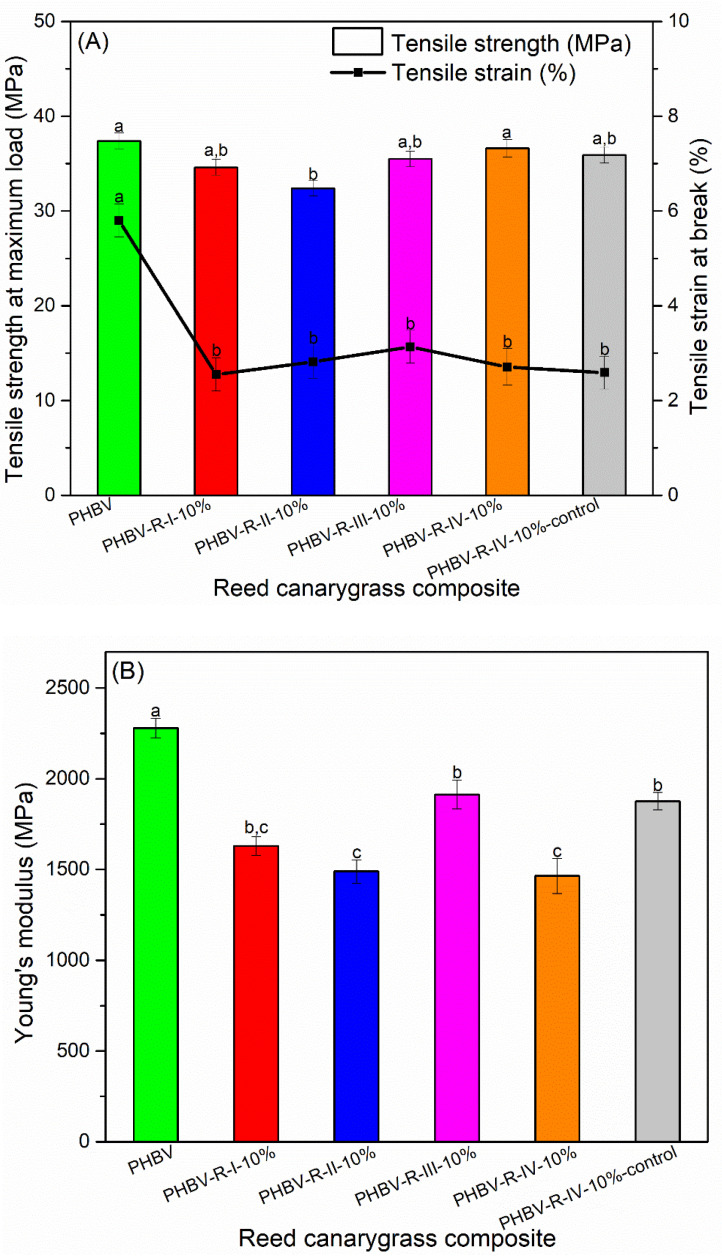

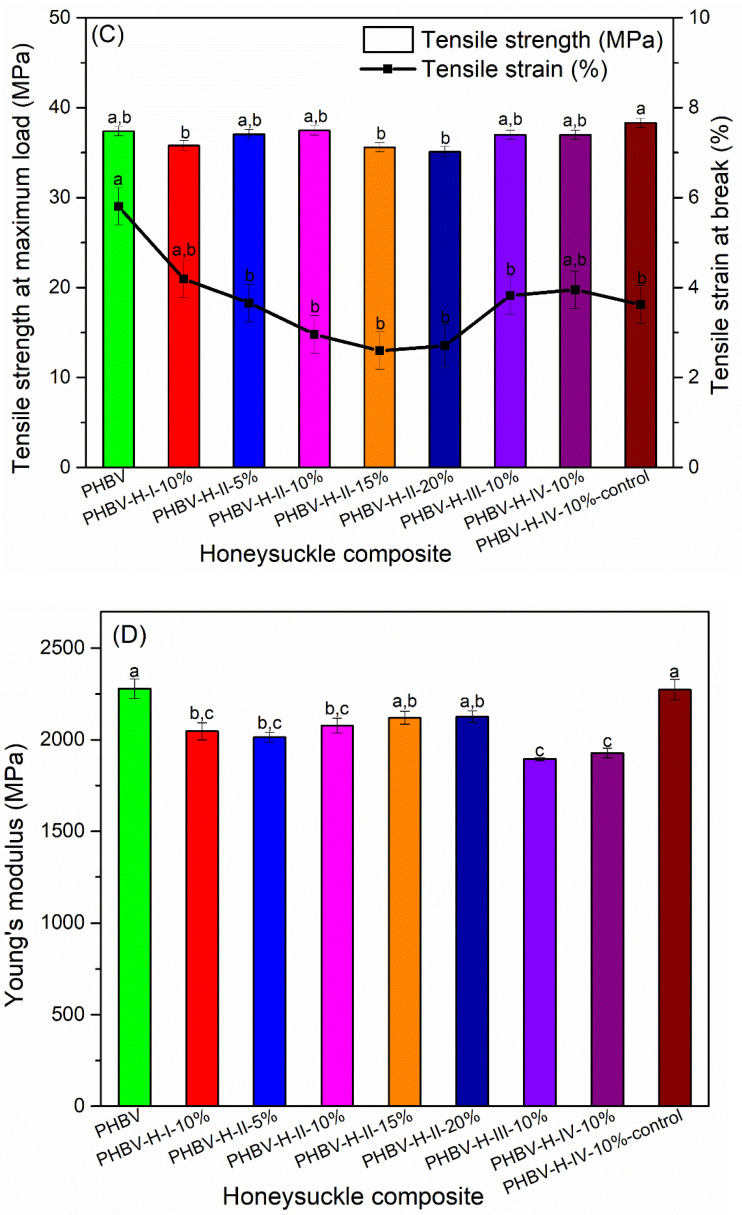
(**A**) Tensile strength and tensile strain of PHBV and its composites with RC fibers. (**B**) Young’s modulus of PHBV and its composites with RC fibers. (**C**) Tensile strength and tensile strain of PHBV and its composites with HS fibers. (**D**) Young’s modulus of PHBV and its composites with HS fibers. Means showing different letters are significantly different at α = 0.05.

Addition of 10% *v*/*v* NaOH-treated RC fibers and 10–20% *v*/*v* NaOH-treated HS fibers (fiber particle size ≤ 600 µm) did not significantly affect PHBV tensile strength (Figure 9A,C, expect for PHBV-C-II-10% which had a slightly lower tensile strength than PHBV) but lowered the tensile strain. It has been reported that the decreasing trend in the PHBV tensile strength was caused by the de-wetting effect of the fibers: when an external force is applied, stress concentration around the fiber particles in the fiber/matrix interface region occurs, and fibers debone from the matrix, forming weak points in the composites and, therefore, decreasing the composite strength [70]. This de-wetting effect usually becomes more predominant with increasing fiber loading [70]. In the current PHBV/fiber composites, the lack of the effect on PHBV tensile strength as well as no change in composite strength with increased fiber loading indicated improved fiber–matrix compatibility.

Interestingly, although alkali treatment increased the fiber–PHBV interfacial bond, the enhanced fiber–PHBV compatibility did not result in any improvement in the composite tensile properties. Similar results were observed in previous research on composites of unsaturated polyester resin and NaOH (10%)-treated hemp fibers [57]. The lack of the improvement in composite tensile strength was attributed to the reduction of the fiber tensile properties after alkali treatment [57]. In our case, as discussed in Section 3.3, although alkali treatment increased fiber tensile strength, alkali treatment decreased fiber tensile strain, resulting in decreased fiber tensile toughness, which may affect the composite tensile properties. Moreover, the effect of the fiber addition on restricting PHBV crystallization, as discussed in DSC analysis, can contribute to the decrease in the PHBV matrix tensile strength. Lastly, the composites had decreased tensile strain compared with PHBV, which was probably due to the low fiber tensile strain.

While the composite tensile strength is more dependent on the matrix tensile strength and the fiber/matrix compatibility, tensile modulus is influenced more by the fiber impregnation and fiber aspect ratio [71]. Addition of 10% *v*/*v* RC fibers (both treated and untreated) decreased PHBV tensile modulus (Figure 9B), with no obvious fiber size dependence. PHBV/RC composites with alkali-treated fibers had lower modulus than that with untreated fibers due to the reduction in fiber modulus caused by the alkali treatment, as discussed in Section 3.3. Addition of 15–20% *v*/*v* alkali-treated (10%-5h NaOH treatment) HS fibers with particle size of 297–425 μm and 10% *v*/*v* untreated fibers with particle size ≤600 µm had no significant effect on PHBV modulus, while other PHBV/HS composites had lower modulus than PHBV (Figure 9D). Like RC fibers, the modulus decreasing trend caused by the HS fiber addition was not dependent on fiber particle size. This result is different from previous studies where addition of miscanthus (untreated) fibers increased PHBV modulus from approximate 1.2 to 4.5 GPa [71,72], probably due to their composites having higher fiber loading (30%), larger fiber particle size (average diameter 0.29 mm and length 2.07 mm), and higher fiber modulus (~6 GPa). The decreased composite modulus in this study was probably caused by the low fiber modulus and the reduced PHBV crystallinity up on fiber addition [73].

To summarize, addition of the alkali-treated RC and HS fibers did not significantly affect PHBV tensile strength but lowered the tensile strain as well as the tensile modulus. The decreased tensile modulus and stiffness made the PHBV/plant fiber composites more suitable for applications where some flexibility is desired, such as semirigid food packaging.

## 4. Conclusions

Green composites from PHBV bioplastic and invasive plant-derived fibers were developed. The effect of the NaOH treatment on the mechanical and thermal properties of the fibers and the composites was investigated. The optimal NaOH treatment for the maximum fiber–PHBV interfacial bond and fiber strength was obtained. In addition, the effect of the fiber size, fiber loading, and fiber type, i.e., nonwood plant fiber (reed canarygrass fiber) and wood fiber (honeysuckle fiber), on the composite mechanical and thermal properties was studied. The value-added use of the invasive plant fibers could help solve the ecological problems, ensure environmental and economic benefits, and improve the sustainability of the composite manufacturing industry. The new biocomposites perform similarly to some of the conventional plastics and, with reduced cost and improved thermal stability, they are promising bio-alternatives to conventional plastics for a variety of industrial applications, such as lightweight body panels for automobiles. Our research on PHBV composites with fibers derived from two typical different invasive plant sources can also provide baseline data for future research on plastic/plant fiber composites.

## Figures and Tables

**Table 1:** Barrel temperatures of PHBV/fiber composite fabrication via extrusion.

Heaters	Temperatures (°C)
1 (Below hopper)	180
2	175
3	175
4	170
5	170
6	160
7	160
8	155
9	150
10 (Die)	145

**Table 2:** Composites from PHBV bioplastic and invasive plant-derived fibers.

Sample Name	Composite Composition	Fiber Particle Size
PHBV	PHBV (processed under the same condition for the biocomposite preparation)	N/A
PHBV-R-I-10%	PHBV + 10 % *v*/*v* RC * fiber with optimal NaOH treatment (2%, 3 h) ***	425–600 μm
PHBV-R-II-10%	PHBV + 10 *v*/*v* % RC fiber with optimal NaOH treatment (2%, 3 h)	297–425 μm
PHBV-R-III-10%	PHBV + 10 *v*/*v* % RC fiber with optimal NaOH treatment (2%, 3 h)	250–297 μm
PHBV-R-IV-10%	PHBV + 10 *v*/*v* % RC fiber with optimal NaOH treatment (2%, 3 h)	<250 μm
PHBV-R-IV-10%-control	PHBV + 10 *v*/*v* % untreated RC fiber	<250 μm
PHBV-H-I-10%	PHBV + 10 *v*/*v* % HS ** fiber with optimal NaOH treatment (10%, 5 h)	425–600 μm
PHBV-H-II-5%	PHBV + 5 *v*/*v* % HS fiber with optimal NaOH treatment (10%, 5 h)	297–425 μm
PHBV-H-II-10%	PHBV + 10 *v*/*v* % HS fiber with optimal NaOH treatment (10%, 5 h)	297–425 μm
PHBV-H-II-15%	PHBV + 15 *v*/*v* % HS fiber with optimal NaOH treatment (10%, 5 h)	297–425 μm
PHBV-H-II-20%	PHBV + 20 *v*/*v* % HS fiber with optimal NaOH treatment (10%, 5 h)	297–425 μm
PHBV-H-III-10%	PHBV + 10 *v*/*v* % HS fiber with optimal NaOH treatment (10%, 5 h)	250–297 μm
PHBV-H-IV-10%	PHBV + 10 *v*/*v* % HS fiber with optimal NaOH treatment (10%, 5 h)	<250 μm
PHBV-H-IV-10%-control	PHBV + 10 *v*/*v* % untreated HS fiber	<250 μm

* RC: reed canarygrass, ** HS: honeysuckle, *** Optimal NaOH treatment for the two fibers were determined by evaluating the mechanical performance of the treated fibers and the fiber–matrix bond.

## Data Availability

The authors confirm that the data supporting the findings of this study are available within the article.
